# Preoperatively Predicting Risk Stratification for GISTs ≤2 cm by Radiomics Model: A Dual-center Study

**DOI:** 10.2174/0115734056419448251211063018

**Published:** 2026-01-27

**Authors:** Ri-Jiang Wu, Yan Tan, Zhi-Xing Zhang, Yao Feng, Yu-Mei Wu

**Affiliations:** 1 Department of Medical Imaging, Shanxi Medical University, Shanxi Provincial People’s Hospital Affiliated to Shanxi Medical University, Taiyuan, Shanxi Province, China; 2 First Hospital of Shanxi Medical University, Taiyuan, Shanxi Province, China; 3 Department of Radiology, Shanxi Provincial People’s Hospital Affiliated to Shanxi Medical University, Taiyuan, Shanxi Province, China; 4 Department of Pathology, Shanxi Provincial People’s Hospital Affiliated to Shanxi Medical University, Taiyuan, Shanxi Province, China; 5 Department of Medical Imaging, Shanxi Medical University, Taiyuan, Shanxi Province, China

**Keywords:** Gastrointestinal stromal tumors, Computed tomography, Risk stratifications, Radiomics, Nomogram

## Abstract

**Introduction:**

Small gastrointestinal stromal tumors (SGISTs, maximum diameter≤2 cm) still carry a risk of malignancy, and their preoperative evaluation remains a significant challenge. Radiomics, an emerging technique for analyzing image data, has yet to be employed to assess the risk stratification of SGISTs. To develop and validate a CT radiomics model for the preoperative prediction of risk stratification in patients with SGISTs.

**Method:**

This study enrolled 133 patients with SGISTs, including 97 in the low-grade group and 36 in the high-grade group. Patients were randomly assigned to a training set (n = 93) and a testing set (n = 40) at a ratio of 7:3. Radiomics features were extracted from preoperative CT images, and dimensionality reduction was performed using the LR-LASSO to identify the most predictive features for constructing the radiomics model. Clinical features were evaluated using univariate and multivariate logistic regression analyses to develop a clinical model. Subsequently, the optimal radiomics and clinical features were integrated to establish a combined model. Model performance was evaluated using ROC curve analysis, and a corresponding nomogram was generated to facilitate clinical application. The Delong test was used to compare the ROC curves, with a *p*-value < 0.05 considered statistically significant.

**Results:**

Univariable clinical analysis identified maximal tumour diameter as the only significant predictor, with the clinical model achieving an AUC of 0.641 (95% CI: 0.533–0.748). Among the radiomics signatures derived from multiphase CT (non-contrast to delayed phases), the model based on portal venous phase images demonstrated the highest discriminative ability, yielding the best AUC values in both the training set (AUC = 0.848, 95% CI: 0.764–0.931) and the testing set (AUC = 0.824, 95% CI: 0.696–0.953). The combined model, which integrated radiomics features with maximum tumour diameter, demonstrated improved performance, attaining an AUC of 0.862 (95% CI: 0.743–0.975) in the training set and 0.859 (95% CI: 0.743–0.975) in the testing set. Notably, the predictive performance of both the radiomics and combined models was significantly greater than that of the clinical model (DeLong test, P < 0.05). However, no statistically significant differences were observed between the AUC values of the radiomics and combined models. Calibration curves indicated a good fit, and the DCA demonstrated that both the radiomics model and the combined model provided greater clinical benefits.

**Discussion:**

The radiomics model demonstrated superior performance to the clinical model for the preoperative prediction of risk stratification in SGISTs. As a visualization tool, the nomogram of the combined model plays a critical role in optimizing early surgical resection decisions.

**Conclusion:**

The radiomics model could serve as an effective tool for non-invasive risk stratification of SGISTs, offering clear advantages over risk stratification models based solely on conventional clinical parameters. This approach could support improved preoperative clinical decision-making.

## 1 INTRODUCTION

Gastrointestinal stromal tumors (GISTs) are the most common mesenchymal tumors of the gastrointestinal tract. They can occur anywhere along the GI tract but are most common in the stomach (50%), small bowel (25%), colon/rectum (10%), and esophagus (5%) [1, 2]. The NIH-modified risk schema stratifies GISTs into four tiers (very low-high) based on: 1) location, 2) maximal diameter, 3) mitotic index, and 4) rupture status. This system effectively predicts recurrence, metastasis, and therapeutic response. GISTs with a diameter of ≤2 cm and a mitotic rate >5 per 50 high-power fields (HPFs) carry a certain risk of recurrence and metastasis [3, 4]. Small GISTs with maximum diameter≤2 cm (SGISTs) may harbor malignant potential, and should therefore be promptly considered for surgical resection [5, 6]. Therefore, preoperative assessment of risk stratifications of SGISTs is crucial for guiding clinical decisions regarding surgical resection [7].

Current preoperative diagnostic modalities face significant challenges. The NIH classification system of GISTs relies on postoperative histopathological evaluation, inherently limiting its utility for preoperative planning [3, 4]. Although endoscopic ultrasound-guided fine needle biopsy (EUS-FNB) and aspiration (EUS-FNA) enable tissue sampling [8, 9], the limited specimen volume often precludes reliable mitotic count assessment and comprehensive risk evaluation [10]. Conventional imaging parameters (tumor size, CT characteristics) contribute to risk stratification [11-13] but remain constrained by interpretive subjectivity, observer bias, and limited capacity to address tumor heterogeneity.

Radiomics has emerged as a transformative approach in oncologic imaging, leveraging advanced texture analysis and machine learning to decode tumor biology through quantitative feature extraction. This methodology has proven effective in tumor characterization, staging, and outcome prediction across multiple malignancies [11, 14, 15]. Radiomics has shown potential in GISTs risk stratification [14, 11], but its application in SGISTs has been scarcely reported.

This study aims to develop a CT radiomics model to preoperatively predict the risk stratification of SGISTs for the first time, with the goal of optimizing therapeutic planning and providing potential guidance for early surgical resection decisions.

## 2 MATERIALS AND METHODS

### 2.1 Subjects

This retrospective, dual-center study was granted an exemption by the Institutional Review Board and complied with the Declaration of Helsinki. We initially screened 593 patients from two medical centers (Center 1 and Center 2) who underwent curative-intent surgery for primary gastric gastrointestinal stromal tumors (GISTs) between January 2017 and August 2024. The inclusion criteria were: (1) preoperative contrast-enhanced CT performed within 15 days before surgery; (2) availability of complete clinicopathological data.The exclusion criteria were: (1) receipt of neoadjuvant therapy; (2) preoperative or intraoperative tumor rupture; (3) maximum tumor diameter >2 cm; (4) poor image quality; (5) indeterminate pathological grading. After applying these criteria, a total of 133 patients were enrolled. Center 1 contributed 95 cases (high-grade: low-grade = 13:82) and Center 2 contributed 38 cases (high-grade: low-grade = 23:15). According to the modified NIH criteria [4], high-grade tumors (n=97) had >5 mitoses/50 HPFs, while low-grade (n=36) had ≤5. These 133 patients were then randomly allocated into a training set (n=93; high-grade: low-grade = 25:68) and a testing set (n=40; high-grade: low-grade = 11:29) at a ratio of 7:3. Fig. (**1A**) outlines the patient selection flowchart.

### 2.2 Patient Clinical Characteristics and Clinical Model Establishment

Clinical factors and CT findings included Age, Gender, Maximum diameter (cm), Location (gastric or others), Necrosis (present or absent), Homogeneous enhancement (absent or present), Intra-tumoral vessel (absent or present), Calcification (present or absent), Growth patterns (Endoluminal, Exophytic, and Mixed), and CT value. Mean CT attenuation was quantified in four phases: non-contrast, arterial, venous, and delayed. The above data were summarized in Table **1**. Univariate and multivariate regression were performed to identify independent clinical and CT predictors for clinical prediction rule derivation.

Image acquisition employed these CT systems: GE Revolution APEX, SOMATOM Force, and PHILIPS iCT. After an 8-hour fast, patients orally ingested 800-1000 mL of water to optimize gastric distension before the examination. Scanning was performed with these parameters:1.5-5 mm slice thickness, pitch 0.9, 120 kV, and 300 mAs. As part of the diagnostic protocol, all enrolled participants underwent comprehensive CT imaging, including both non-contrast and contrast-enhanced scans. Patients received intravenous iohexol (300 mg I/mL) at 3.5-4.0 mL/s (total volume: 70-100 mL). Arterial phase scanning commenced at 30 seconds post-injection, followed by venous and delayed phases at 60 and 120 seconds, respectively. CT images were transferred to the PACS for interpretation at the workstations. Two abdominal radiologists (14 and 5 years of experience) separately reviewed all images. Any discrepancies were resolved through joint re-evaluation to establish a consensus diagnosis. Both readers remained blinded to the pathological results.

### 2.3 Development of Radiomics Models

#### 2.3.1 Image Preprocessing

Before image segmentation, it is essential to preprocess images to reduce the data heterogeneity arising from different CT devices. First, the voxel size should be resampled to 1×1×1 mm^3^, and the anisotropic images should be homogenized to minimize the influence of voxel size on radiomics features. The voxel intensity values were discretized using a fixed bin width of 25 HU to reduce image noise and standardize intensities, ensuring consistent intensity resolution across all images. Additionally, images were normalized, with signal intensities scaled to a range of 1 to 500 HU to minimize variations in signal intensity of images acquired by different machines.

### 2.4 Tumor ROI Delineation

Manual segmentation of the GISTs on axial images was conducted by a radiologist (5 years of experience) using ITK-SNAP (version 4.0.0, http://www.itksnap.org/). A senior radiologist with 14 years of experience reviewed the delineations and made modifications as necessary. Delineation was performed on each CT slice. Both radiologists were unaware of the patients' risk stratification. To determine intra-observer agreement using the intraclass correlation coefficient (ICC), repeat segmentations were performed one month later on a randomly selected cohort of 30 patients.

### 2.5 Feature Extraction and Feature Selection

Radiomic features were extracted from both non-contrast and contrast-enhanced CT images using FeAture Explorer (FAE, V 0.3.6), which integrates the PyRadiomics library. Through PyRadiomics, ten categories of features were generated: Original, Wavelet-transformed, Square, Square Root, Laplacian of Gaussian, Logarithmic, Exponential, Gradient, Local Binary Pattern (2D), and Local Binary Pattern (3D). Each category comprised five feature matrices: first-order (18 features), Gray Level Co-occurrence Matrix (GLCM, 22 features), Gray Level Size Zone Matrix (GLSZM, 16 features), Gray Level Run Length Matrix (GLRLM, 16 features), and Gray Level Dependence Matrix (GLDLM, 22 features; 14 features for the original category). The Original category also included an additional feature matrix (with 14 features). Further details on the radiomic features can be found at https://pyradiomics.readthedocs.io/en/latest/features.html.

### 2.6 Radiomics Models Construction

Post-extraction, features demonstrating good observer consistency (ICC≥0.75) were selected and normalized using a z-score. To mitigate dataset imbalance, SMOTE generated synthetic samples *via* minority class interpolation, equilibrating class frequencies. The feature space was standardized using z-score normalization (mean-centered). Pearson correlation coefficients screened features to reduce dimensionality prior to model development. Dimensionality reduction produced decorrelated features in the transformed space. Feature selection combined analysis of variance with recursive feature elimination (RFE). Classification employed: SVM, random forests, LASSO-penalized logistic regression, and decision tree classifiers. Five-fold cross-validation was employed during model training. These workflows were conducted using FAE and R software (version 4.2.3) (http://www.R-project.org). The process is shown in Fig. (**1B**). The performance of the radiomics models was assessed using receiver operating characteristic (ROC) curve analysis. Additionally, the accuracy, sensitivity, specificity, positive predictive value (PPV), negative predictive value (NPV), and Youden index were calculated for each model.

### 2.7 Development of the Combined Model

The most relevant clinical factors were combined with the Rad-score to create a combined model using logistic regression, and a nomogram was subsequently generated. The DeLong test was used to compare AUC values between the clinical model, radiomics model, and combined model. DCA evaluated clinical utility, while calibration curves assessed model calibration.

### 2.8 Statistical Analysis

The clinical characteristics of patients were compared by analyzing percentages for categorical data and means with standard deviations for continuous data. The comparison was performed using the χ2 or Fisher's exact test for categorical data, and t tests for continuous data. Radiomics features were extracted from preoperative contrast-enhanced CT images using standardized segmentation protocols. Dimensionality reduction was performed using Logistic Regression-Least Absolute Shrinkage and Selection Operator (LR-LASSO) to identify optimal predictive features. The selected features were then used to construct the radiomics signature (Rad-score). Clinical variables associated with tumor grade were screened using univariate logistic regression. Multivariate logistic regression with backward stepwise selection was then used to identify independent predictors for the clinical model. A combined model was developed by integrating the Rad-score and selected clinical features. Model performance was evaluated using receiver operating characteristic (ROC) curve analysis, with the area under the curve (AUC) measuring discriminative ability. Calibration was assessed through calibration curves and Hosmer-Lemeshow tests, and a nomogram was generated to visualize the combined model. The Delong test was used to compare the AUCs of the radiomics, clinical, and combined models. All statistical tests were two-sided, with P<0.05 considered statistically significant. Analyses were performed using R software (version 4.2.0; packages: glmnet, rms, pROC). The Intra-class Correlation Coefficient (ICC) was calculated to assess intra-reader agreement for radiomic feature extraction, with an ICC value≥0.75 indicating good consistency.

## 3 RESULTS

### 3.1 Patient Clinical Characteristics and Clinical Model Establishment

The clinical characteristics of the patients in both the training and testing datasets are shown in Table **1**. Univariate analysis identified that the maximum diameter of the tumor was the only parameter demonstrating a statistically significant association (P<0.05). No significant differences were found in age, gender, margin, contour, location, necrosis, homogeneous enhancement, calcification, or growth patterns. The maximal tumor diameter served as the basis for a clinical prediction model developed through logistic regression. The clinical model yielded an AUC of 0.641 (95% CI, 0.533-0.748) in the training set, and 0.705 (95% CI, 0.535-0.876) in the testing set. Table **2** presents diagnostic performance metrics: sensitivity, specificity, accuracy, PPV, and NPV.

### 3.2 Developing and Evaluating Radiomics Models

Computer-generated randomization allocated datasets into training (70%) and testing (30%) subsets. The training cohort comprised 93 cases (68 positive, 25 negative), while the testing set comprised 40 cases (29/11=positive/negative). A total of 1316 radiomic features were extracted from the volume of interest of each lesion using portal venous phase imaging. Following inter-observer consistency evaluation, 1181 features demonstrating excellent reproducibility (ICC≥0.75) were retained for subsequent model development. The radiomic feature selection process yielded five optimal predictors, whose coefficient distributions are graphically summarized in Fig. (**2**). The radiomics model demonstrated robust performance in both the training set (AUC = 0.848, 95% CI: 0.764-0.931) and the testing set (AUC = 0.824, 95% CI: 0.696-0.953), shown in Fig. (**3A** and **B**).

Original_Shape_Sphericity quantifies tumor roundness, with higher values generally suggesting benign lesions, whereas irregular shapes may indicate malignancy or aggressive growth. Logarithm_glszm_SmallAreaHighGray LevelEmphasis (SAGE) measures the prominence of small high-intensity areas post-log transformation, with higher values indicating tissue heterogeneity associated with aggressive tumors, necrosis, or hypoxia. Squareroot_glszm_SizeZone NonUniformityNormalized assesses zone-size variability after square-root transformation and normalization, with higher values indicating tumor heterogeneity and irregular growth patterns indicative of invasive phenotypes. Wavelet-HL_glcm_MCC evaluates pixel correlation in the HL wavelet subband, wherein lower values reflect disorganized tissue and potential malignancy. Wavelet-LL_glszm_SizeZoneNonUni formity quantifies zone-size non-uniformity in the LL subband, with higher values associated with tissue heterogeneity, aggressive tumors, or fibrous stroma.

Table **S1** and Fig. (**S1**) comprehensively tabulate performance metrics for radiomics models across multiphase CT (non-contrast to delayed) and fused data, including AUC-ROC, diagnostic accuracy indices, and predictive values. Statistical comparisons of AUC values between models were performed using the DeLong test. Additionally, Table **2** details the sensitivity, specificity, accuracy, Youden index, PPV, and NPV for each individual model in both training and testing sets.

### 3.3 Developing and Evaluating a Combined Model

The combined model demonstrated good performance in both the training set (AUC = 0.862, 95% CI: 0.743-0.975) and the testing set (AUC = 0.859, 95% CI: 0.743-0.975) as shown in Fig. (**3A,B**). The predictive performance of both the radiomics and combined models was significantly superior to that of the clinical model (Delong test, P<0.05), with increases in AUC of 0.014 (training set) and 0.035 (testing set), respectively. Although the combined model improved the performance compared with the radiomics model, these improvements were not statistically significant (P > 0.05). The decision curve analysis showed that both the radiomics model and the combined model provided a higher net benefit in the training set Fig. (**3C,D**), while the calibration curve demonstrated good agreement between predicted and observed outcomes Fig. (**3E,F**). A predictive nomogram integrating radiomics scores and maximum diameter was developed to calculate individualized risk scores for SGISTs in Fig. (**4**), and the schematic representation of the nomogram is displayed in Fig. (**5**).

## 4 DISCUSSION

This study demonstrates that CT radiomics enables preoperative risk stratification in small gastrointestinal stromal tumors (SGISTs ≤2 cm). We developed and validated three predictive models, including the radiomics model, clinical model, and combined model, for SGISTs risk stratification. Notably, both the radiomics and combined models significantly outperformed the clinical model in predictive accuracy.

Univariate analysis identified the maximum tumour diameter as the only independent clinical predictor for SGISTs risk stratification, indicating that larger tumour size is associated with greater malignant potential, a finding consistent with previous research outcomes [4, 11]. However, the clinical model yielded only limited discrimination (AUC = 0.641), reflecting inherent limitations in conventional metrics. Notably, in intermediate- to high-risk SGISTs, arterial phase vascular signs, intratumoral necrosis, and ulceration showed no statistically significant differences compared with the low-risk group. Thisfindingisconsistentwith the observations by Jia *et al*. [16], but contrasts with previous studies on larger GISTs [17], where these imaging features were more strongly associated with malignant potential. We propose that the limited tumour volume in sub-2 cm lesions may reduce susceptibility to metabolic stressors (*e.g*., ischaemia, nutritional deprivation), thereby diminishing necrosis and subsequent ulceration. These observations collectively suggest that conventional clinical parameters offer limited predictive value for SGISTs malignancy potential. This aligns with the findings of studies conducted by Ulusan *et al*. [18].

The portal venous phase-derived radiomics signature emerged as the most robust predictor (training AUC = 0.848, testing AUC = 0.824; DeLong test P < 0.05 vs. other phases), aligning with prior evidence [16, 19] highlighting portal venous phase radiomic features as key determinants of tumour heterogeneity in GISTs. According to Wei *et al*. [20], the prolonged retention of contrast agents in malignant lesions during the portal venous phase may be attributed to KIT-activating mutations, which promote increased endothelial permeability. In addition, the portal venous phase improves the delineation of tumour boundaries with higher accuracy, enhances lesion visibility, and facilitates precise segmentation of tumour targets. These characteristics collectively establish portal venous phase imaging as a powerful tool in radiomics, improving the accuracy of tumour detection and analysis.

The choice of scanning phase has long been a topic of debate in radiomics-based risk stratification research for GISTs. However, Sun *et al*. [21], in their investigation of malignancy risk prediction in 2–5 cm GISTs, proposed that radiomic features derived from arterial-phase imaging can accurately differentiate the malignant potential of GISTs. A potential explanation is that smaller lesions may lack well-developed neovascular networks, resulting in less distinct arterial-phase features.

In summary, the portal venous phase is the optimal window for assessing heterogeneity in GISTs, benefitting from the unique vascular permeability dynamics and enhanced morphological delineation characteristic of this phase. Future studies could optimise radiomics-based risk stratification systems for SGISTs by integrating multiphase features or establishing correlation models between tumour biological characteristics and imaging phases. Such multidimensional integration would not only help resolve current controversies regarding phase selection but also overcome the limitations of single-phase biological characterisation, thereby providing more comprehensive imaging biomarkers to advance precision diagnosis and treatment.

Feature selection for the radiomics model identified five discriminative features: two wavelet transform features, one logarithm transform feature, one square root transform feature, and a single feature extracted from the source images. Therefore, we propose that wavelet, Gaussian, and square root transforms enhance detail visibility in original images, revealing additional information that better reflects inter-tumoral heterogeneity-consistent with the findings of Zhao *et al* [22]. GLCM is an image analysis technique that characterizes the spatial arrangement and distribution of pixel intensities through a grey-level co-occurrence matrix [23]. GLCM and GLSZM may offer a comprehensive view of tissue characteristics [24]. Among the final retained radiomics features, texture-based parameters predominated (GLCM: n=1; GLSZM: n=3). This indicates that second-order features characterize spatial and intensity relationships between pixels than first-order features, enabling deeper assessment of tumor heterogeneity and improved discriminative capacity [20]. Of the five radiomics features ultimately selected in this study, three were derived from GLSZM. These features quantify the heterogeneity and variation in the distribution of grayscale values within the image [25], and all showed a positive correlation with the Redscore. Nomogram analysis indicated that an elevated Redscore is associated with an increased risk of high-grade SGIST. Furthermore, an increase in the Redscore was accompanied by enhanced texture irregularity and greater internal complexity of the volume of interest (VOI), suggesting more pronounced intratumoral heterogeneity in SGIST-positive cases [26].

The combined model incorporating both radiomics signatures and maximum diameter of tumor achieved slightly improved performance (AUC=0.862 in training set, AUC=0.859 in testing set). Although the combined model yielded slightly higher AUC values than the radiomics model in the training (0.862 vs. 0.848) and testing (0.859 vs. 0.824) sets, these differences were not statistically significant. The slight AUC gain (0.014) in the combined model, despite incorporating maximum tumor diameter, underscores the predominant contribution of radiomic features. Unlike previous GIST studies focusing on larger lesions [14], our study provides essential reference information for clinicians to predict the risk stratification of SGISTs using a nomogram. In our radiomics model, Rad-score values were significantly correlated with pathological grade, as shown in Fig. (**5**). As a visualization tool for the combined model, the nomogram plays a critical role in optimizing early surgical resection decisions. Its minimal reliance on complex molecular analyses ensures it is practically applicable in community healthcare settings with limited resources.

## 4.1 Limitations and Implications

Although our study highlights the predominance of radiomics in risk stratification, three limitations warrant emphasis: (1) Despite applying data harmonization procedures (normalization and balancing), heterogeneity in CT scanners across two medical centers may impact the stability of radiomic features. Therefore, multicenter validation using standardized imaging protocols is crucial to confirm the translational relevance of these findings. (2) The use of tumor size, a conventional clinical metric, may not sufficiently capture multidimensional tumor biology, highlighting the need to explore alternative clinical-pathological synergies. (3) Future studies should focus on integrating functional imaging biomarkers or molecular profiles such as KIT and PDGFRA mutations to augment clinical-radiomic fusion models. (4) This study did not include external validation. We plan to incorporate external validation in future research to further enhance the robustness and generalizability of the model.

## CONCLUSION

This study primarily introduces a CT radiomics model for the preoperative prediction of risk stratification in patients with SGISTs. The radiomics model could serve as an effective tool for the non-invasive risk stratification of SGISTs, offering significant advantages over assessments based solely on conventional clinical parameters. As a visualization tool for the combined model, the nomogram plays a critical role in optimizing decisions regarding early surgical resection. It should be noted that we plan to incorporate external validation in future research to further enhance the model's robustness and generalizability.

## RESEARCH PERSPECTIVES

Future research should aim to validate the model with a larger sample size and incorporate additional integral factors, such as KIT and PDGFRA mutations.

## Figures and Tables

**Fig. (1):**
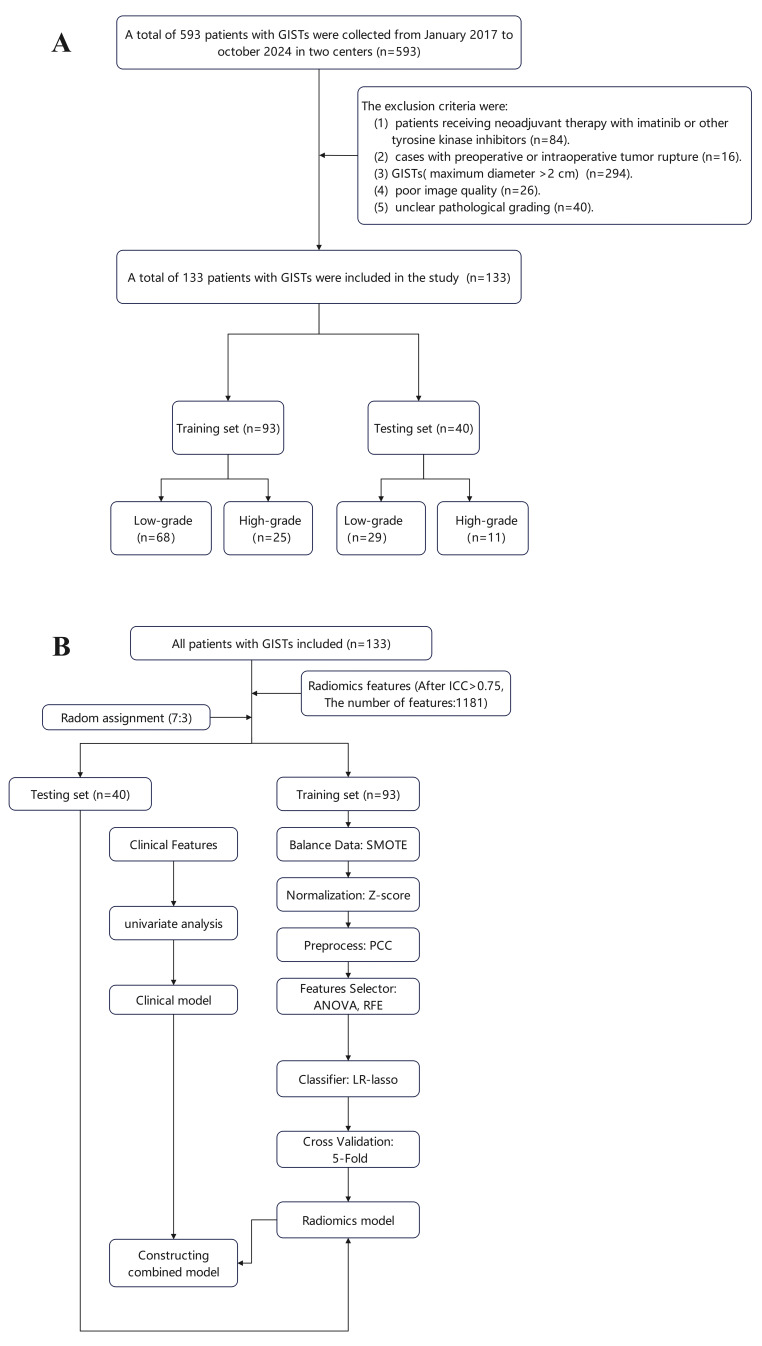
Patient screening and enrollment flowchart (**A**) and Radiomics analysis workflow (**B**).

**Fig. (2):**
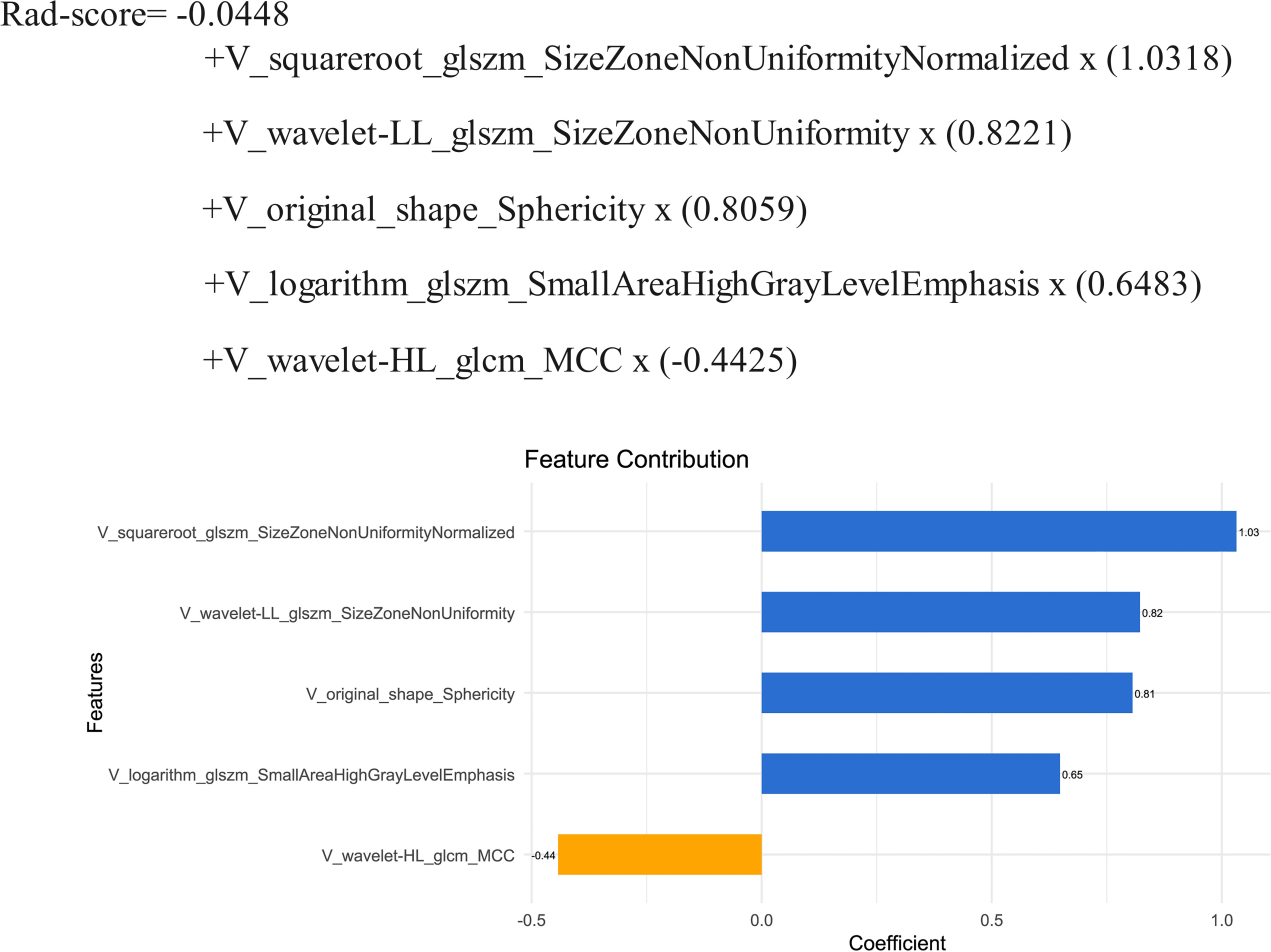
Rad-score and the coefficient distributions of Radiomic features.

**Fig. (3):**
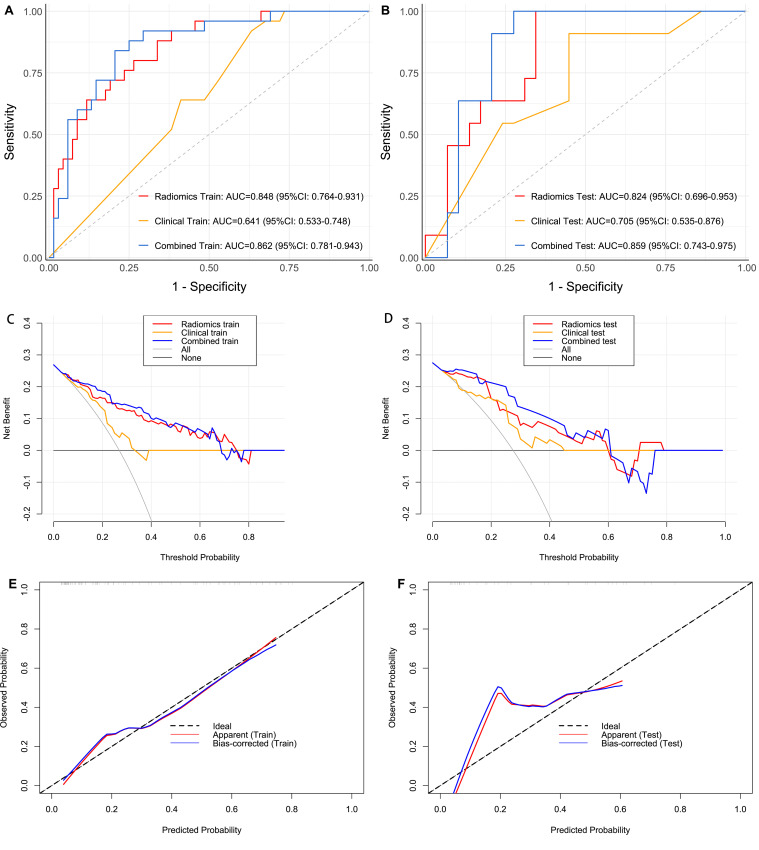
ROC curves for the Radiomics model, Clinical model, and Combined model in the training and testing sets (**A**-**B**). DCA curves for the Radiomics model, Clinical model, and Combined model in the training and testing sets (**C**-**D**). Calibration curves for the Radiomics model in the training and testing sets (**E**-**F**).

**Fig. (4):**
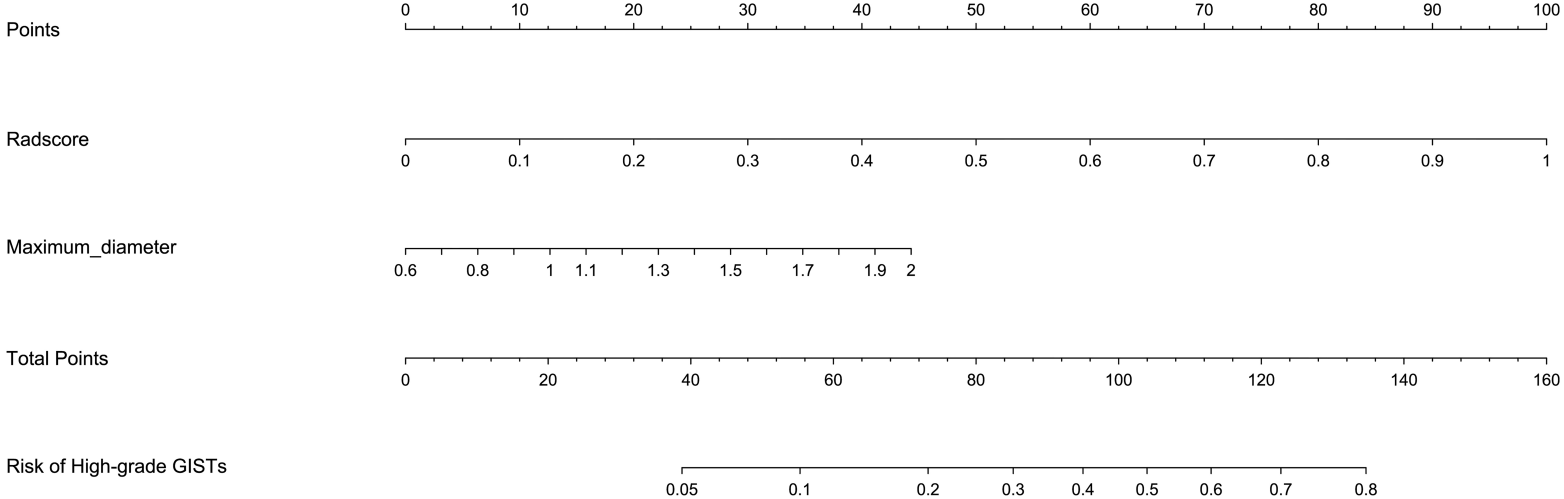
Nomogram for predicting the risk of high-grade GISTs.

**Fig. (5):**
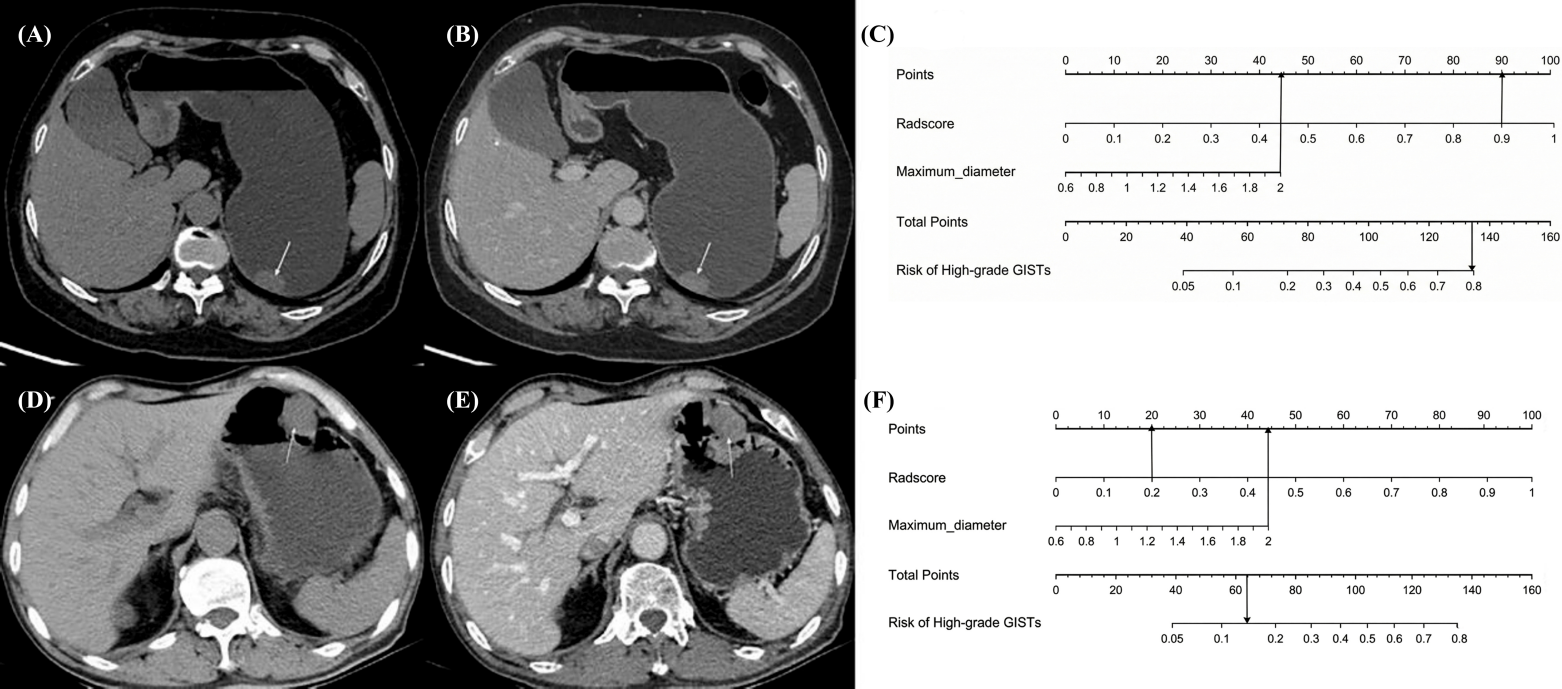
Nomogram schematics (**A**-**C**): For Rad-score=0.903 and max diameter=2.0 cm, malignancy probability=0.798. Histologically confirmed intermediate-risk stratification. Nomogram schematics (**D**-**F**): For Rad-score = 0.201 and max diameter = 2.0cm, malignancy probability= 0.142. Histologically confirmed low-risk stratification.

**Table 1:** Clinical characteristics of patients with GISTs in the training and testing datasets.

**Characteristics**	**Training Dataset (n = 93)**			**Testing Dataset (n =40)**			
	Low-grade (n = 68)	High-grade (n = 25)	*p*-value	Low-grade (n = 29)		High-grade (n = 11)	*p*-value
**Age in years**	58.63±9.65	61.60±7.88	0.172	55.14±6.28		53.00±5.85	0.334
**Gender (n, %)**			0.099				0.927
**Male**	**32(34.4)**	**7(7.5)**		**11(27.5)**		**4(10.0)**	
**Female**	36(38.7)	18(19.4)		18(45.0)		7(17.5)	
**Maximum diameter(cm)**	1.60(1.10-2.00)	2.00(1.50-2.00)	0.031*	1.40(1.05-1.95)		2.00(1.60-2.00)	0.048*
**Location (n, %)**			0.292				0.533
**Gastric**	66(70.1)	23(24.70)		28(70.0)		11(27.5)	
**Others**	2(2.6)	2(2.6)		1(2.5)		0(0)	
**Growth patterns (n, %)**			0.243				0.529
**Endoluminal**	41(44.1)	12(12.9)		17(42.5)		5(12.5)	
**Exophytic**	11(11.8)	8(8.6)		2(5.0)		2(5.0)	
**Mixed**	16(17.2)	5(5.4)		10(25.0)		4(10.0)	
**Contour (n, %)**			1.000				0.479
**Regular**	65(69.8)	24(25.8)		28(70.0)		10(25.0)	
**Irregular**	3(3.2)	1(1.2)		1(2.5)		1(2.5)	
**Margin (n, %)**			0.269				-
**Well-defined**	68(73.1)	24(25.8)		29(72.5)		11(97.2)	
**Poorly**	0(0)	1(1.1)		0		0	
**Necrosis (n, %)**			0.058				0.479
**Absent**	67(72.0)	22(23.7)		28(70.0)		10(25.0)	
**Present**	1(1.1)	3(3.2)		1(2.5)		1(2.5)	
**Calcification (n, %)**			0.075				0.043
**Absent**	57(61.3)	25(26.9)		20(50.0)		11(27.5)	
**Present**	11(11.8)	0(0.0)		9(22.5)		0(0)	
**Homogeneous enhancement (n, %)**			0.107				0.039
**Absent**	8(8.6)	7(7.5)		2(3.1)		4(10.0)	
**Present**	60(64.5)	18(19.4)		27(69.4)		7(17.5)	
**Intra-tumoral vessel (n, %)**			0.269				0.275
**Absent**	68(71.3)	24(25.8)		29(72.5		10(25.0)	
**Present**	0(0)	1(2.9)		0(0)		1(2.5)	
**Plain CT value(HU)**	38.00(33.00-41.75)	33.00(29.50-41.00)	0.165	37.00(32.50-41.00)		36.00(33.00-40.00)	0.743
**Arterial phase CT value(HU)**	57.00(49.00-64.00)	51.00(41.00-64.00)	0.104	54.00(42.00-58.50)		53.00(48.00-66.00)	0.530
**Venous phase CT value (HU)**	69.00(61.00-83.00)	68.00(55.50-79.00)	0.296	65.00(57.50-79.50)		70.00(56.00-79.00)	0.835
**Delayed phase CT value(HU)**	76.00(66.50-91.75)	71.00(60.00-80.50)	0.138	70.00(64.00-86.50)		72.00(55.00-82.00)	1.000

Note:*Statistically significant, P < 0.05. The Mann-Whitney test was used to analyze continuous variables with non-normal distribution, whereas continuous variables with a normal distribution were examined using the t-test. For categorical variables, either the χ^2^ test or Fisher's exact test was employed.

**Table 2:** Predictive performance of each model for GISTs grading in the training and testing sets.

**Model**	**Set**	**AUC (95%CI)**	**Accuracy**	**Youden Index**	**Sensitivity**	**Specificity**	**PPV**	**NPV**
Radiomics	training	0.848(0.764-0.931)	0.720	0.542	0.880	0.662	0.489	0.938
	testing	0.824(0.696-0.953)	0.750	0.655	1.000	0.655	0.524	1.000
Clinical	training	0.641(0.533-0.748)	0.516	0.288	0.920	0.368	0.348	0.926
	testing	0.705(0.535-0.876)	0.650	0.461	0.909	0.552	0.435	0.941
Combined	training	0.862(0.781-0.943)	0.807	0.634	0.840	0.794	0.600	0.931
	testing	0.859(0.743-0.975)	0.875	0.828	1.000	0.828	0.688	1.000

## Data Availability

The data and supportive information are available within the article.

## LIST OF ABBREVIATIONS

GISTs: Gastrointestinal stromal tumors
NCCN: National Comprehensive Cancer Network
GLCM: Gray level co-occurrence matrix
GLDM: Gray level dependence matrix
GLRLM: Gray level run length matrix
GLSZM: Gray level size zone matrix
LASSO: Least absolute shrinkage and selection operator
LRLASSO: Logistic regression *via* LASSO
LR: Logistic regression
PCC: Pearson correlation coefficient
SMOTE: Synthetic Minority Over-sampling Technique
NPV: Negative predictive value
PPV: Positive Predictive Value
RFE: Recursive feature elimination
ROC: Receiver operating characteristic
SVM: Support vector machine
RF: Random Forest
AUC: Area under the curve
CI: Confidence interval

## STANDARDS OF REPORTING

STROBE guidelines were followed.
