# Supplementary material for: Preoperatively Predicting Risk Stratification for GISTs ≤2 cm by Radiomics Model: A Dual-center Study
Source: Curr Med Imaging. 2026 Jan 27;22:e15734056419448. doi: 10.2174/0115734056419448251211063018 (PMC13463225; doi:10.2174/0115734056419448251211063018)
Supplement: Supplementary file 1 [file CMIM-22-E15734056419448_SD1.pdf]

1875-6603/26

Send Orders for Reprints to reprints@benthamscience.net

BENTHAM SCIENCE

CrossMark

Current Medical Imaging

Content list available at: https://benthamscience.com/journals/cmir

Supplementary Material

Preoperatively Predicting Risk Stratification for GISTs ≤2 cm by Radiomics Model: A Dual-center Study

Ri-Jiang Wu[1], Yan Tan[2,*], Zhi-Xing Zhang[3], Yao Feng[4] and Yu-Mei Wu[5]

[1]Department of Medical Imaging, Shanxi Medical University, Shanxi Provincial People's Hospital Affiliated to Shanxi Medical University, Taiyuan, Shanxi Province, China
[2]First Hospital of Shanxi Medical University, Taiyuan, Shanxi Province, China
[3]Department of Radiology, Shanxi Provincial People's Hospital Affiliated to Shanxi Medical University, Taiyuan, Shanxi Province, China
[4]Department of Pathology, Shanxi Provincial People's Hospital Affiliated to Shanxi Medical University, Taiyuan, Shanxi Province, China
[5]Department of Medical Imaging, Shanxi Medical University, Taiyuan, Shanxi Province, China

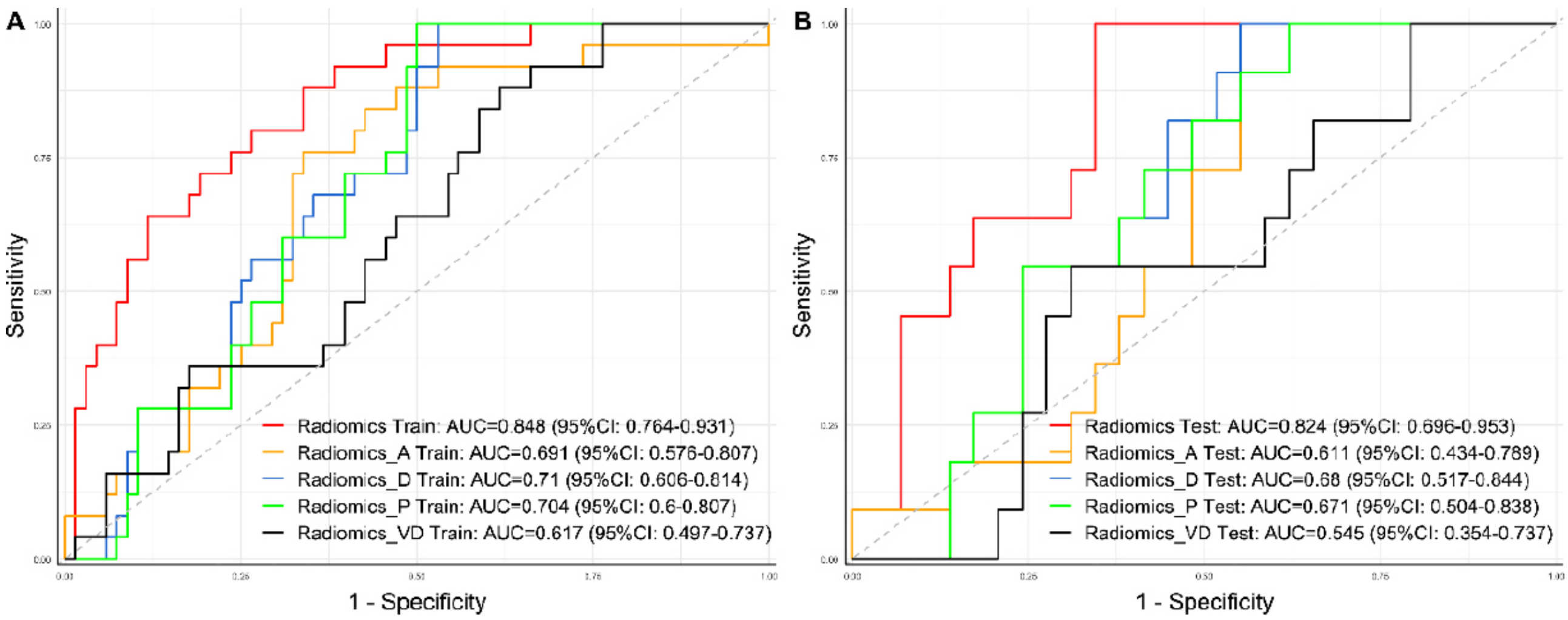

Fig. (S1). ROC curves for the Radiomics model, Radiomics_A model, Radiomics_D model, Radiomics_P model, and Radiomics_VD model in the training and testing sets (**A-B**).

Table S1. Predictive performance of each model for GISTs grading in the training and testing sets.

Model	Set	AUC (95%CI)	Accuracy	Youden Index	Sensitivity	Specificity	PPV	NPV
Radiomics	training	0.848(0.764-0.931)	0.720	0.542	0.880	0.662	0.489	0.938
	testing	0.824(0.696-0.953)	0.750	0.655	1.000	0.655	0.524	1.000
Radiomics A	training	0.691(0.577-0.805)	0.688	0.422	0.760	0.662	0.452	0.882
	testing	0.611 (0.437-0.786)	0.550	0.379	1.000	0.379	0.379	1.000
Radiomics P	training	0.704(0.600-0.807)	0.634	0.500	1.000	0.500	0.424	1.000
	testing	0.671(0.506-0.836)	0.550	0.435	1.000	0.435	0.379	1.000
Radiomics D	training	0.710(0.607-0.813)	0.807	0.471	1.000	0.471	0.410	1.000

DOI: 10.2174/0115734056419448251211063018, 2026, *22*, e15734056419448

Model	Set	AUC (95%CI)	Accuracy	Youden Index	Sensitivity	Specificity	PPV	NPV
	testing	0.680(0.519-0.842)	0.600	0.448	1.000	0.448	0.407	1.000
Radiomics VD	training	0.617(0.498-0.736)	0.516	0.262	0.880	0.382	0.344	0.897
	testing	0.545(0.358-0.733)	0.650	0.235	0.545	0.690	0.400	0.800

© 2026 The Author(s). Published by Bentham Science Publisher.

This is an open access article distributed under the terms of the Creative Commons Attribution 4.0 International Public License (CC-BY 4.0), a copy of which is available at: https://creativecommons.org/licenses/by/4.0/legalcode. This license permits unrestricted use, distribution, and reproduction in any medium, provided the original author and source are credited.

HOW TO CITE:
Wu R, Tan Y, Zhang Z, Feng Y, Wu Y. Preoperatively Predicting Risk Stratification for GISTs ≤2 cm by Radiomics Model: A Dual-center Study. Curr Med Imaging, 2026; 22: e15734056419448.
http://dx.doi.org/10.2174/0115734056419448251211063018
